# Rapid diagnosis of new and relapse tuberculosis by quantification of a circulating antigen in HIV-infected adults in the Greater Houston metropolitan area

**DOI:** 10.1186/s12916-017-0952-z

**Published:** 2017-11-01

**Authors:** Jia Fan, Hedong Zhang, Duc T. Nguyen, Christopher J. Lyon, Charles D. Mitchell, Zhen Zhao, Edward A. Graviss, Ye Hu

**Affiliations:** 1 School of Biological and Health Systems Engineering, Virginia G. Piper Biodesign Center for Personalized Diagnostics, The Biodesign Institute, Arizona State University, Tempe, AZ 85287 USA; 2 Department of Nanomedicine, Houston Methodist Research Institute, Houston, TX 77030 USA; 3 Department of Pathology and Genomic Medicine, Houston Methodist Research Institute, Houston, TX 77030 USA; 4 University of Miami, Leonard M. Miller School of Medicine, Miami, FL 33136 USA; 5 Department of Laboratory Medicine, Clinical Center, National Institutes of Health, Bethesda, MD 20892 USA

**Keywords:** HIV, *Mycobacterium tuberculosis*, Co-infection, Diagnosis, Blood test, CFP-10, Peptide biomarker, Immunoprecipitation, LC-MS/MS

## Abstract

**Background:**

HIV-associated immune defects inhibit tuberculosis (TB) diagnosis, promote development of extrapulmonary TB and paucibacillary pulmonary TB cases with atypical radiographic features, and increase TB relapse rates. We therefore assessed the diagnostic performance of a novel assay that directly quantitates serum levels of the *Mycobacterium tuberculosis* (*Mtb*) virulence factor 10-kDa culture filtrate protein (CFP-10) to overcome limitations associated with detecting *Mtb* bacilli in sputum or tissue biopsies.

**Methods:**

This study analyzed HIV-positive adults enrolled in a large, population-based TB screening and surveillance project, the Houston Tuberculosis Initiative, between October 1995 and September 2004, and assigned case designations using standardized criteria. Serum samples were trypsin-digested and immunoprecipitated for an *Mtb*-specific peptide of CFP-10 that was quantified by liquid chromatography-mass spectrometry for rapid and sensitive TB diagnosis.

**Results:**

Among the 1053 enrolled patients, 110 met all inclusion criteria; they included 60 tuberculosis cases (12 culture-negative TB), including 9 relapse TB cases, and 50 non-TB controls, including 15 cases with history of TB. Serum CFP-10 levels diagnosed 89.6% (77.3–96.5) and 66.7% (34.9–90.1) of culture-positive and culture-negative TB cases, respectively, and exhibited 88% (75.7–95.5) diagnostic specificity in all non-TB controls. Serum antigen detection and culture, respectively, identified 85% (73.4–92.9) and 80.0% (67.3–88.8) of all 60 TB cases.

**Conclusions:**

Quantitation of the *Mtb* virulence factor CFP-10 in serum samples of HIV-infected subjects diagnosed active TB cases with high sensitivity and specificity and detected cases missed by the gold standard of *Mtb* culture. These results suggest that serum CFP-10 quantitation holds great promise for the rapid diagnosis of suspected TB cases in patients who are HIV-infected.

**Electronic supplementary material:**

The online version of this article (doi:10.1186/s12916-017-0952-z) contains supplementary material, which is available to authorized users.

## Background

Tuberculosis (TB) continues to be associated with high morbidity and mortality, particularly in people living with HIV in TB endemic areas, who have a 20- to 30-fold increased risk of developing TB. The World Health Organization (WHO) now estimates that 12% of new TB cases are HIV-positive [1], and that TB is the leading cause of HIV/AIDS-related mortality, accounting for one in three HIV deaths [2]. In regions with high HIV prevalence, most notified cases of tuberculosis are smear-negative or sputum-scarce (the patient is unable to produce sputum) [3]. Delayed or missed TB diagnosis is an important cause of excess mortality in HIV-affected patients, particularly for cases with smear-negative pulmonary and extrapulmonary TB (PTB and EPTB) [4], which are found with increased incidence in this group, and autopsy studies reveal that there is a high proportion of undiagnosed TB in this population [5, 6]. HIV-positive patients also demonstrate higher rates of relapse TB cases due to treatment failure, the emergence of drug resistance, or reinfection with a new *Mtb* strain after successful treatment completion [7, 8]. In regions of high TB incidence, HIV-infected individuals exhibit reinfection rates of 30–50%. Further complicating this situation, frontline TB diagnostics, including acid-fast bacilli (AFB) smear, mycobacterial culture, and Xpert *Mycobacterium tuberculosis* (MTB)/resistance to rifampin (RIF) assays all exhibit reduced sensitivity with paucibacillary TB samples (*Mtb* culture-negative or smear-negative), which are frequently associated with HIV-positive TB cases [9, 10]. There is thus an urgent unmet need for rapid, quantitative, non-sputum-based biomarker tests that can accurately diagnose new and relapse TB cases in clinically challenging patient populations, including HIV-infected subjects [11].

*Mtb* antigen detection provides direct evidence of infection, and circulating *Mtb* antigen levels should be relatively insensitive to pulmonary vs. extrapulmonary infection sites. However, enzyme-linked immunosorbent assays (ELISAs) designed to detect the *Mtb* antigens Immunogenic protein MPB64 (MPB-64) in blood, 6 kDa early secretory antigenic target (ESAT-6) in cerebrospinal fluid, and lipoarabinomannan in urine have not demonstrated adequate diagnostic performance, likely due to low antigen levels, host protein interactions, or homology with non-tuberculous mycobacteria (NTM) antigens [12, 13]. To address these issues, we have developed and published approaches that specifically and sensitively quantify *Mtb* culture filtrate protein 10 (CFP-10) levels in trypsin-digested clinical samples [14, 15]. CFP-10 was chosen for this assay since its secretion promotes immunopathologic host responses, while its deficiency markedly attenuates *Mtb* virulence [16–19], implying that its detection in serum is diagnostic for all active TB cases, including EPTB and paucibacillary PTB [20]. In brief, we have identified a tryptic peptide that distinguishes *Mtb* CFP-10 from its NTM homologs and used polyclonal antibodies raised against this peptide to enrich it from trypsin-digested serum samples for quantitation [15]. In this study, we employed an immunoaffinity-based parallel reaction monitoring (iPRM) liquid chromatography mass spectrometry (LC-MS) assay that used MS/MS fractionation to confirm the target peptide identity for increased specificity. We validated the diagnostic performance of this approach with archived serum samples from patients with HIV enrolled in the Houston Tuberculosis Initiative (HTI), a large population-based TB surveillance study, and found that this assay demonstrated robust sensitivity and specificity for both new and relapse TB cases.

## Methods

### Study population

Study samples and patient data were obtained from HIV-diagnosed subjects enrolled in the HTI between October 1995 and September 2004. The HTI project was a population-based study that prospectively collected data and samples from individuals with clinically suspected and/or laboratory-diagnosed TB cases (positive *Mtb* culture) in Houston, Texas and Harris County. Inclusion and exclusion criteria of the HTI parent study have been described elsewhere [21]. Briefly, the HTI active surveillance study prospectively enrolled all TB suspects reported to the City of Houston Tuberculosis Control Office. The study enrolled 93% of all reported TB patients and 85% of all culture-positive TB patients in the Houston area from October 1995 through September 2004. Trained contact investigators interviewed participants and their non-TB family members to collect demographic and socioeconomic data, medical history, and clinical information to identify TB risk factors. HTI personnel administered a standardized questionnaire to collect demographic, living condition, social contact, incarceration history, drug and alcohol use, and medical history information. Medical and public health records from the City of Houston Department of Health and Human Services clinics and hospitals were obtained for all patients to capture past medical history, including HIV status, TB-related symptoms, chest X-rays, smear and culture results, and previous TB diagnosis history and treatment outcomes. HTI investigators excluded all individuals who did not provide written consent or participate in the interview.

For the current study, we excluded all patients who were HIV-negative or had unknown HIV status, were lost to follow-up, or lacked archived blood samples drawn prior to within 1 month of the anti-TB treatment initiation. TB and non-TB cases were defined by clinical and/or assay criteria for TB diagnosis according to the Tuberculosis (TB) (*Mycobacterium tuberculosis*) 2009 Case Definition by Centers for Disease Control and Prevention (CDC). TB cases (*n* = 60) were classified as culture-positive (*n* = 48) or culture-negative (*n* = 12), which were defined by symptom improvement after anti-TB treatment as assessed by CDC guidelines [22]. Non-TB controls consisted of HIV patients referred to the clinic or doctor as a TB suspect and ultimately designated as non-TB cases by the referral TB physicians after rigorous clinical and laboratory evaluation. Nine of a total of 60 TB cases were relapse TB cases, and 15 of a total of 50 non-TB controls were cured TB cases. HTI subjects were queried against rosters of TB cases periodically received from Texas Department of State Health Services (DSHS) to determine if any subjects previously classified as non-TB cases had developed active disease or if any of the cured TB cases had developed a subsequent TB episode. This indirect, follow-up surveillance continued through October 2011, so that all enrolled persons were subject to at least 7 years of follow-up from the time of sample collection. Both relapse TB and cured TB cases had cures documented by two consecutive negative culture results more than 12 months prior to the collection of the serum samples analyzed in this study [23]. Relapse TB cases presented with new TB cases more than 12 months after these cures, while cured TB cases did not demonstrate TB symptoms at the time of their analyzed blood draw. Culture-positive patients received monthly follow-ups, at minimum, until achieving two consecutive culture-negative results. Culture-negative TB cases were evaluated by chest X-ray (posterior-anterior and lateral) and/or clinical appraisal, according to disease manifestation [22]. One culture-negative TB case revealed a single AFB-positive histology result with a notation that the sample contained rare bacilli. We were unable to determine from the available retrospective data whether this was a smear-positive case with an abnormal culture-negative result or a culture-negative case with a smear-positive result arising from a contamination event or other artifact. We therefore omitted this patient when calculating iPRM sensitivity for smear-positive and smear-negative TB cases. Study protocols were reviewed and approved by the Institutional Review Boards at Houston Methodist Hospital, TX, USA (Pro0002302, Pro00000546, Pro00005327) and the Baylor College of Medicine and Affiliate Hospitals (H-16562). The current study adheres to the Standards for Reporting of Diagnostic Accuracy Studies (STARD) guidelines.

### Study procedures

Clinical procedures performed at enrollment included a medical history, physical examination, blood draw, chest X-ray, mycobacterial specimen collection, and an HIV test (ELISA with western blot confirmation) if HIV status was unknown. AFB smear, *Mtb* culture, and HIV and CD4+ T cell assays were performed by the clinical laboratories of the referring physicians as part of their routine care. Serum samples collected by the HTI team at the time of diagnosis were archived at –80 °C. Serum samples from all TB cases were drawn prior to or within 1 month of the anti-TB treatment initiation, to match the WHO’s definition of a new patient as someone who has never been treated for TB or has taken anti-TB drugs for less than 1 month [24]. All TB patients had paired *Mtb* culture results. Serum samples from non-TB cases were collected after these subjects consented to participate in the study and completed an interview with study staff members.

### iPRM assay procedure

Protein A-conjugated Dynabeads (Thermo Fisher Scientific) were incubated with custom polyclonal antibody (GL Biochem) raised against the *Mtb* CFP-10 target peptide for 2 h (5 μg antibody/300 μg beads per sample) at room temperature, washed with phosphate-buffered saline (PBS)/0.05% Tween-20, and resuspended in PBS/0.05% Tween-20 at a concentration of 30 μg/μL. Serum samples (100 μL) were diluted with 400 μL of 100 mM ammonium bicarbonate, then microwave digested [15], spiked with 1 nmol/L stable-isotope-labeled internal standard peptide (m/z 1603.60; GenScript) matching the CFP-10 target peptide sequence (m/z 1593.75), and mixed with 10 μL prepared antibody-labeled beads for 1 h at room temperature. Beads were then washed with PBS/0.05% Tween-20 and incubated with 1% formic acid (pH < 2.0) to elute bound peptides. Eluates were loaded on a C18 trap column, eluted onto a C18 analytical column, and fractionated with a 0.3 μL/min acetonitrile/formic acid gradient (5–40%) and analyzed using the PRM Mode on a nano-LC UltiMate 3000 high performance liquid chromatography (HPLC) system coupled with an LTQ Velos Pro mass spectrometry system (Thermo Fisher Scientific) (Additional file 1: Figure S1 and Additional file 2: Table S1). Skyline software version 3.5.0.9319 (MacCoss Lab Software) was used to analyze serum MS and MS/MS spectra against a library produced using recombinant CFP-10 digests. Standard curves were generated by spiking healthy donor serum with 0–20 nmol/L recombinant CFP-10 and converting experimental sample MS intensity ratios to absolute molar concentrations by substitution into these calibration curves. Limits of detection (LOD) and quantification (LOQ) were obtained from the mean of each blank plus three times (LOD, 21 pmol/L) or ten times (LOQ, 41 pmol/L) the standard deviation of their noise.

### Statistical analysis

Calculation of median, interquartile range (IQR), sensitivity and specificity, data normality, analysis of variance (ANOVA) with post-test correction (Dunn’s test), Mann-Whitney, and chi-square tests were performed with GraphPad Prism software (version 7.01).

## Results

**Fig. 1:**
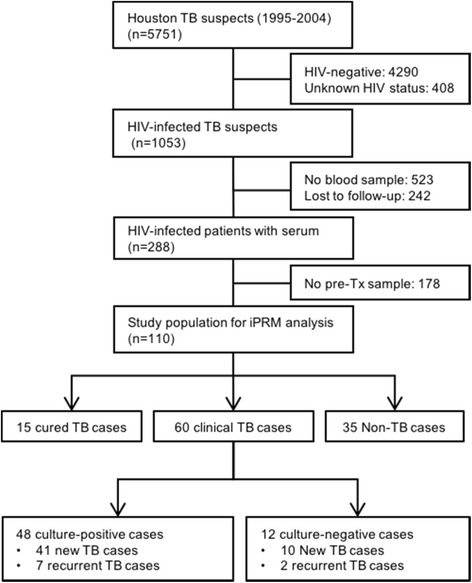
Study flow diagram

Survey of the HTI records identified 1053 eligible HIV-positive HTI subjects, but 701 lacked blood samples and 242 were lost to follow-up, yielding a study population of 110 subjects (Fig. 1). This group contained 60 TB cases confirmed by mycobacterial culture or clinical findings (54.5%), including 9 relapse TB cases; 50 non-TB controls, including 15 cases with history of previously TB (cured TB, 13.6%), who remained TB-negative at analysis; and 35 patients without evidence of TB disease and no TB history (31.8%). The majority (*n* = 26, 74%) of the 35 non-TB patients without a history of TB disease had clinical findings that were initially consistent with a suspected TB case, including fever, cough, chills, or hemoptysis (*n* = 9, 26%); pneumonia (*n* = 7, 20%), *Pneumocystis carinii* pneumonia (*n* = 5, 14%), or atypical pneumonia (*n* = 2, 6%); or chest radiograph abnormalities (*n* = 3, 9%). The remaining subjects revealed symptoms not consistent with suspect TB cases, including renal failure (*n* = 3, 9%) or individual cases (*n* = 6, 17%) of hepatitis C, hepatitis B, cryptococcal meningitis, fever with lymphadenopathy, fever with altered mental status, or seizure. The 15 non-TB patients with a prior TB history (cured TB cases) represented subjects enrolled with a TB case who had more than a year of follow-up after their initial TB cure and did not present with any TB-associated symptoms at the time of their subsequent analyzed blood draw.

Study participants were primarily middle-aged adults (median 39 years of age), male (74.5%), and African American (65.5%), but none of these factors appreciably varied with TB status (Table 1). CD4^+^ T cell counts did not differ between non-TB and TB cases or culture-positive and culture-negative TB cases, and HIV levels were similar among TB and non-TB cases, but higher in culture-positive than in culture-negative TB cases.

**Table 1:** Demographics and clinical characteristics of the study participants

	Total patients	All non-TB controls (*N* = 50)	All TB cases (*N* = 60)	TB cases		
				Culture-positive (*n* = 48)	Culture-negative (*n* = 12)	*p* value
Age, years (IQR)^a^	39 (34–46)	41 (34–46)	37 (34–48)	37 (34–48)	38 (33–48)	0.470^b^/0.978^c^
Sex, male (%)^a^	82 (74.5)	36 (72.0)	46 (76.7)	35 (72.9)	11 (91.7)	0.662^b^/0.262^c^
Race: no. (%)^a^						0.749^b^/0.713^c^
Black	72 (65.5)	36 (72.0)	36 (60.0)	28 (58.3)	8 (66.7)	
White	16 (14.5)	5 (10.0)	11 (18.3)	10 (20.8)	1 (8.3)	
Hispanic	20 (18.2)	8 (16.0)	12 (20.0)	9 (18.8)	3 (25.0)	
Asian or Pacific Islander	2 (1.8)	1 (2.0)	1 (1.7)	1 (2.1)	– (–)	
CD4 T cells/μL (IQR)^a^	110 (39–320)	127 (56–358)	99 (35–280)	73 (35–257)	237 (16–458)	0.364^b^/0.260^c^
Log^10^ HIV copies/mL (IQR)^a^	5.3 (4.6–5.6)	5.4 (4.6–5.7)	5.1 (4.3–5.6)	5.5 (5.0–5.8)	3.9 (2.8–5.5)	0.309^b^/0.002^c^
TB disease site: no. (%)^a^						0.013^c^
PTB	30 (50.0)		30 (50.0)	20 (41.7)	10 (83.3)	
EPTB	37 (33.6)		9 (15.0)	7 (14.9)	2 (16.7)	
PTB and EPTB	47 (42.7)		21 (35.0)	21 (46.8)	– (–)	
History of previous TB: no. (%)^a^	24 (21.8)	15 (30.0)	9 (15.0)	7 (14.6)	2 (16.7)	0.060^b^/> 0.999^c^
AFB smear positive: no. (%)^a^	13 (21.7)		12 (20.0)	12 (25.0)	1 (8.3)^d^	–
CXR cavitary lesions: no. (%)^a^	14 (23.3)		14 (23.3)	12 (31.6)	2 (23.3)	0.542^c^

Data, no. (% or IQR)

*IQR* interquartile range, *AFB* acid-fast bacilli, *CXR* chest X-ray, *PTB* pulmonary TB, *EPTB* extrapulmonary TB

^a^Percentage or interquartile range of corresponding column population

^b^ *p* value of Student *t* test, Mann-Whitney *U* test, or chi-square test for difference between All non-TB controls and All TB cases

^c^ *p* value of chi-square test for difference between culture-positive and culture-negative TB cases

^d^One culture-negative TB case was marked AFB-positive with a “rare bacilli” notation

Patients with TB primarily had pulmonary phenotypes, with 30 PTB (50%), 21 PTB/EPTB cases (35%), and 9 EPTB cases (15%). Most TB cases were culture-positive (48 of 60; 80%), which were similarly split among PTB (41.7%) and PTB/EPTB (46.8%) cases, and new (80.4%; 41 of 51) and relapse (77.8%; 7 of 9) TB cases. *Mtb* culture detected all PTB/EPTB cases, 77.8% of the EPTB cases, and 66.7% of the PTB cases, and most culture-positive PTB and PTB/EPTB cases were determined using non-invasive sputum samples (78.0%; 32 of 41). Extrapulmonary diagnoses for EPTB and PTB/EPTB cases were made primarily with invasive lymph node (42.9%; 12 of 28), pleural fluid (17.9%; 5 of 28), or cerebrospinal fluid (10.7%; 3 of 28) samples (Additional file 1: Figure S2). AFB smear results exhibited 100% specificity but poor sensitivity for both culture-positive (25.0%) and culture-negative (8.3%) TB cases. Radiographic features did not differ between culture-positive and culture-negative cases.

Serum iPRM assay diagnosed 85% (73.4–92.9%) of all the study TB cases (48 culture-positive and 12 culture-negative TB cases) and revealed 88% (75.7–95.5%) diagnostic specificity for all non-TB controls (35 subjects with no history of TB and 15 cured TB cases) (Table 2). In the subgroup analysis (Table 2), iPRM detected culture-positive TB cases with 89.6% (77.3–96.5) sensitivity and had similar sensitivities for culture-positive PTB (90%; 68.3–98.8), EPTB (100%; 59.0–100), and PTB/EPTB (85.7%; 63.7–97.0) cases. Culture-negative TB cases were detected with 66.7% (34.9–90.1) overall sensitivity by iPRM, but the small number of these cases precluded subgroup sensitivity comparisons. The diagnostic sensitivity of iPRM exceeded *Mtb* culture for all PTB (83.3% vs. 66.7%; 25 of 30 vs. 20 of 30, respectively) and EPTB (88.9% vs. 77.8%; 8 of 9 vs. 7 of 9) cases, while culture revealed better sensitivity for PTB/EPTB cases (100% vs. 85.7%; 21 of 21 vs. 18 of 21). Both culture and iPRM results detected relapse TB cases with equal sensitivity (77.8%, 40.0–97.2). Serum CFP-10 detection exhibited 100% sensitivity for culture-positive/smear-positive TB cases but reduced sensitivities for paucibacillary culture-positive/smear-negative (83.3%; 66.5–93.0) and culture-negative/smear-negative (60.0%; 26.2–87.8) cases.

**Table 2:** Sensitivity and specificity of iPRM assay results for indicated study groups and subgroups

		Sensitivity^a^	
		Smear-positive TB cases (*n* = 12)	Smear-negative TB cases (*n* = 47)
	Sensitivity (*n* = 60)		
All TB cases (*n* = 60)	51/60 (85.0%, 73.4–92.9)	12/12 (100%, 73.5–100)	38/47 (80.9%, 66.1–90.6)
Culture-positive TB (*n* = 48)	43/48 (89.6%, 77.3–96.5)	12/12 (100%, 73.5–100)	30/36 (83.3%, 66.5–93.0)
PTB only (*N* = 20)	18/20 (90.0%, 68.3–98.8)	6/6 (100%, 54.1–100)	12/14 (85.7%, 57.2–98.2)
EPTB only (*N* = 7)	7/7 (100%, 59.0–100)	–	7/7 (100%, 59.0–100)
PTB and EPTB (*N* = 21)	18/21 (85.7%, 63.7–97.0)	6/6 (100%, 54.1–100)	12/15 (80.0%, 59.5–98.3)
Culture-negative TB (*n* = 12)	8/12 (66.7%, 34.9–90.1)	–	7/11 (60.0%, 26.2–87.8)
PTB only (*N* = 10)	7/10 (70%, 34.8–93.3)	–	6/9 (66.7%, 29.0–96.3)
EPTB only (*N* = 2)	1/2 (50%, 1.3–98.7)	–	1/2 (50.0%, 1.3–98.7)
Relapse TB cases	7/9 (77.8%, 47.4–99.7)	3/3 (100%, 29.2–100)	4/6 (66.7%, 28.4–99.5)
	Specificity (*n* = 50)		
All non-TB controls (*n* = 50)	44/50 (88.0%, 75.7–95.5)		
No history of previous TB (*N* = 35)	32/35 (91.4%, 76.9–98.2)	–	–
History of previous TB (“cured TB,” *N* = 15)	12/15 (80.0%, 51.9–95.7)	–	–

Data, *n*/*N* (%, 95 confidence interval)

^a^One culture-negative TB case was marked AFB-positive with a “rare bacilli” notation, but since this result could not be confirmed or determined to arise from a contamination event or other artifact, this sample was omitted when calculating iPRM assay sensitivity for smear-positive and smear-negative TB cases

**Fig. 2:**
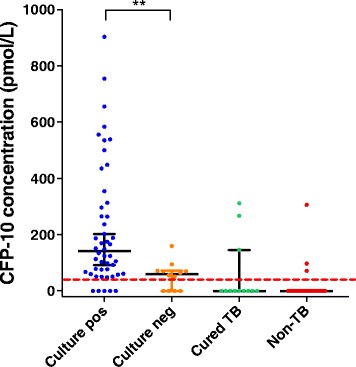
CFP-10 concentrations in culture-positive and culture-negative TB cases, cured TB cases, and non-TB cases. *Solid horizontal lines* indicate the median and 95% confidence intervals for each group, and the *red dashed line* indicates the CFP-10 cut-off value (41 pmol/L) for TB diagnosis. **p < 0.005 by Mann-Whitney *U* test

Smear- and culture-negative results are reflective of reduced mycobacterial concentrations in the assayed samples, which could also reduce serum concentration of CFP-10 to explain the reduced iPRM sensitivity for smear- and/or culture-negative TB cases. We therefore compared serum CFP-10 levels in culture-positive and -negative cases and found that serum CFP-10 was significantly lower in culture-negative vs. culture-positive cases (Fig. 2).

Serum iPRM results respectively exhibited 91.4% (76.9–98.2%) and 80.0% (51.9–95.7%) specificity when samples were taken from patients without and with a previous history of TB disease in non-TB controls (Table 2). However, subsequent review of the three iPRM-positive samples from the patients without a history of TB found results consistent with increased TB risk. For example, one patient with a non-TB diagnosis presented with a cough as the only symptom, but had a 44-mm tuberculin skin test (TST) induration. Another patient had night sweats, fever, fatigue, and weight loss, pleural effusion, lymphadenopathy, plate-like opacification, and close contact with a TB-positive patient, but received a non-TB diagnosis after 3 months of anti-TB treatment. The third subject had multiple TB-associated symptoms, including cough, fever, night sweats, dyspnea and hemoptysis, and right upper lobe posterior segment consolidation with cavity formation consistent with TB or, less likely, necrotizing pneumonia. Similar analyses of the cured TB cases with iPRM-positive results were less informative. One patient had a subsequent pleural effusion, but there were no results available for the thoracentesis fluid or for the cause of death 5 months later; the second patient did not authorize a chart review for TB symptoms following the post-cure blood draw; and the third had no available evidence of subsequent TB-related symptoms.

**Fig. 3:**
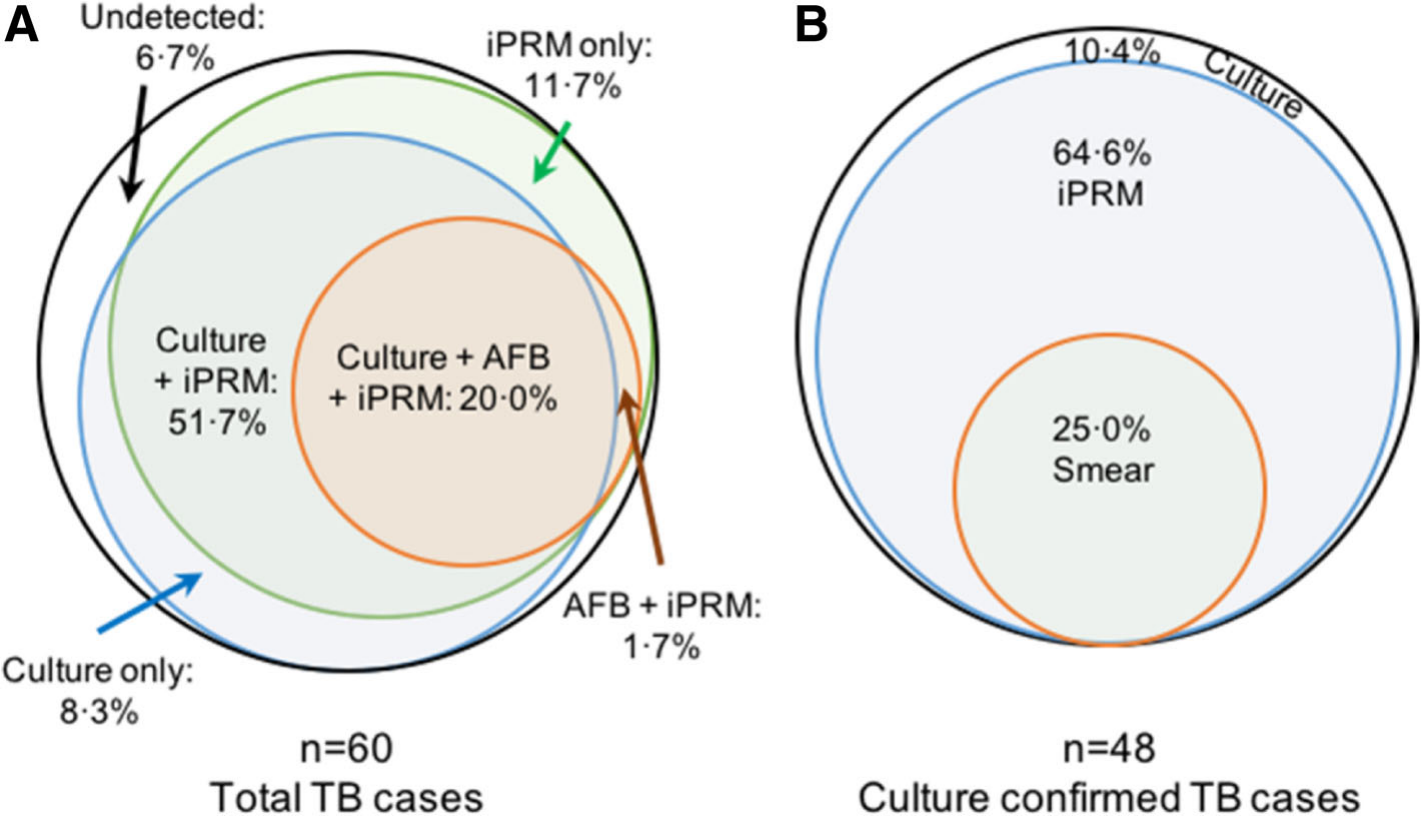
Venn diagrams showing the proportions of patients diagnosed by liquid culture, smear, and iPRM for **a** total TB cases and **b** culture-confirmed TB cases

Culture, AFB, and iPRM recognized 20% (12/60) of all the TB cases in common (Fig. 3a) and missed 6.7% (4/60) of these TB cases, but, upon exclusion of the AFB results, culture and iPRM detected 71.7% (43/60) of all the TB cases in common. Culture and iPRM uniquely classified 8.3% (5/60) and 11.7% (7/60) of all TB cases, and iPRM and AFB, respectively, detected 89.6% (43/48) and 25.0% (12/48) of all culture-positive cases (Fig. 3b).

Rates of relapse TB, normally low in the general population, are significantly elevated in HIV-infected patients. Relapse TB cases accounted for 15% (9 of 60) of all the study TB cases and 37.5% (9 of 24) of cases in patients with a prior TB history (relapse TB and cured TB cases). Relapse cases had similar distribution among culture-positive (14.6%) and culture-negative (16.7%) TB cases, and were detected with equal sensitivity (77.8%, 40.0–97.2%) by culture and iPRM (Tables 1 and 2). Notably, one patient with relapse TB had multiple blood samples available between his two TB episodes, one of which iPRM found to be positive 3 months before his relapse TB diagnosis. Relapse and cured cases revealed similar overall demographics (Additional file 3: Table S2), but more time had elapsed on average from the TB cures of relapse vs. cured TB cases (7 vs. 3 years), although HIV titer and CD4+ T cell count differences suggestive of poorer disease control and immune function in this group were not significant.

## Discussion

This study assessed the ability of a novel assay to diagnose TB cases in an HIV population at high risk for TB. The assay directly quantified CFP-10, an important *Mtb* virulence antigen, in peripheral blood serum samples. CFP-10 demonstrates homology with factors expressed by other mycobacterium species; however, we used trypsin digestion to release a peptide that distinguishes *Mtb* CFP-10 from homologs produced by other mycobacteria upon analysis by mass spectrometry. This digestion should also disrupt protein complexes that could mask serum CFP-10 levels in conventional immunoassays targeting intact *Mtb* proteins.

This iPRM platform fulfills desired WHO characteristics for new TB diagnostics [11]. It analyzes a biomarker rather than bacterial isolates, which can require invasive biopsies and may lack sufficient bacilli for reliable diagnosis. It uses a non-sputum specimen readily obtained from all suspect patient groups, diagnoses PTB and EPTB cases with high sensitivity and specificity, and exhibits good performance in culture-negative patient samples. It also uses a streamlined process amenable to a high-throughput operation to allow rapid TB diagnosis and therapy initiation.

We tested iPRM assay performance in a population recommended for such novel assays, HIV-positive patients at risk for TB disease, and found that it had an 85% overall diagnostic sensitivity, exceeding the WHO-defined 66% optimal sensitivity for new high-priority non-sputum diagnostic tests [11]. Sensitivities for culture-positive PTB cases with smear-positive (100%) and smear-negative (86.1%) results also met or exceeded recommended sensitivity thresholds (98% and 68%) for these cases, as did diagnostic sensitivity for culture-positive EPTB cases (100% iPRM vs. 85% proposed). The iPRM assay exhibited 66.7% sensitivity in the subgroup of clinically diagnosed (culture-negative) TB cases. Clinically diagnosed TB cases are subject to over-diagnosis [25] since the lack of bacteriologic confirmation may cause other infections, including NTM infections, to be misdiagnosed as TB cases. The potential for clinical over-diagnosis may be even higher in HIV-positive patients [26] and can reduce the apparent sensitivity of our iPRM assay in culture-negative vs. culture-positive TB cases in this study. Future study of iPRM performance with culture-negative TB cases is important, because rapid diagnosis of culture-negative TB cases is essential to reduce transmission as well as increased morbidity and mortality in HIV-affected populations prone to culture-negative cases. Such diagnoses can also help to reduce misdiagnosis and inappropriate treatment of non-TB cases with relatively toxic anti-TB treatment regimens.

One limitation of this study is its retrospective design and relatively small number of TB cases, particularly clinical and relapse TB cases, representing the epidemiology of combined TB and HIV infection in a metropolitan area with low TB incidence (ranging from 26 to 46 cases per 100,000 population between 1995 [27] and 2013 [28]). The use of long-archived serum samples may also negatively affect iPRM assay performance due to potential biomarker degradation during long-term storage and an unknown number of freeze-thaw cycles, although long-term follow-up was necessary to permit evaluation of TB recurrence. Study subjects also were selected based on their serum availability, which could potentially introduce unknown bias. Most serum samples (97%) from patients with active TB cases were obtained after treatment initiation due to the recruitment mechanism of the parent study. Cases were treated for a mean of 6.8 days before serum collection, with a median of 9 days and a 25–75% distribution range of 5 to 17 days, although serum draws tended to be later for culture-negative than for culture-positive cases: 14 (6–23) days vs. 9 (5–17) days, respectively. All culture-positive TB cases in this study had a positive culture sample collected at or following their blood draw. Culture-positivity appears to decline gradually (97%, 93%, 89%, to 83% positivity) over the first 4 weeks of anti-TB treatment [29], in agreement with other studies [30, 31] that indicate that *Mtb* infectivity can extend 5–6 weeks after standardized anti-TB therapy initiation. However, reduced *Mtb* abundance in these treated cases still could cause our results to under-report the diagnostic sensitivity of this assay for untreated TB cases. Limited group size may also influence assay sensitivity and specificity, particularly for subgroup analyses, since limited subject numbers may magnify variance.

WHO guidance recommends Xpert MTB/RIF use as an initial diagnostic in HIV-positive patients with suspected TB, although its sensitivity decreases with smear- and culture-negative TB cases, which are commonly found in this population. We found that iPRM exhibited 86.1% (70.5–95.3) sensitivity in smear-negative, culture-positive cases, favorably comparing to Xpert sensitivities of 47.3% (29.2–67.0) to 61.1% (35.7–82.7) reported for such cases in other adult HIV-positive populations [32, 33]. Further, while there is little information for Xpert use in adult culture-negative HIV-positive TB cases, one study reported 9.1% Xpert sensitivity for this group [33], which is markedly less than the 66.7% (34.9–90.1) iPRM sensitivity we observed for these cases.

EPTB accounts for up to 25% of all TB cases and is more common in HIV-positive patients, but *Mtb* culture and Xpert MTB/RIF assays require invasive specimens for EPTB diagnosis. One study found highly variable Xpert sensitivities for lymph node (84%) and cerebrospinal (56%) and pleural fluid (17%) specimens [34]. Another study found smaller sensitivity differences among lymph node biopsy specimens (83%) and pleural (46%) and cerebrospinal (81%) fluid samples [35]. Finally, a third study reported Xpert sensitivities for lymph node biopsy (96%) and pleural fluid (34%) samples but could not estimate sensitivity for cerebrospinal fluid samples [36]. Xpert sensitivity thus appears to vary considerably with biopsy type. There is little information for HIV-mediated effects on Xpert sensitivity with EPTB samples, but one study suggests that in HIV-positive patients Xpert displays reduced diagnostic sensitivity with pulmonary aspirates but enhanced sensitivity with extrapulmonary (pleural, pericardial, and cerebrospinal fluid) specimens [37]. Serum iPRM assays, however, showed similar overall sensitivity for PTB (83.3%) and EPTB (88.9%) cases, which only slightly diverged in culture-positive cases (90% vs. 100%).

Relapse TB poses an additional threat to TB control in HIV-infected populations, which have an approximately 30-fold increased rate of TB relapse [38–40]. We have also reported that HIV infection is independently associated with TB recurrence after successful completion of adequate treatment documented by negative culture results [27]. In this study, 37.5% of HIV-positive patients with a previous TB history had relapse TB cases within a mean of 7 years. The ability of iPRM to rapidly assess all manifestations of TB disease in HIV-positive patient populations thus represents a potentially important advance for TB diagnosis and containment. We calculated that iPRM reagent costs for one sample were less than $10, and thus comparable to the per test consumable costs of the mycobacteria growth indicator tube (MGIT) culture and Xpert assay, which are $8.05 and $11.97, respectively [41]. The “sample-to-answer” time of our iPRM assay is approximately 2 h. This is much faster than traditional liquid/solid mycobacterial culture, which averages 14–21 days for a result and can require as long as 6 weeks, and is comparable to Xpert assays, which can provide report data within 1 day. However, we predict that we can improve both assay costs and response times, since our assay has substantial room for optimization. Automated systems for coupling magnetic beads to mass spectrometry are in routine use for disease diagnosis in clinical laboratories and could allow rapid clinical translation of our iPRM diagnostic approach. MS is now routinely employed by many clinical laboratories to improve the sensitivity and specificity of clinical tests, screen for diseases, monitor drug therapy, analyze peptides and proteins for diagnostic testing, and identify causes of infections for targeted therapies [42]. MS systems are not commonly available in resource-poor areas with high levels of HIV-associated TB disease; however, two different approaches could address this issue. Samples could be processed at remote sites and shipped to central laboratories with MS systems, or they could be analyzed using inexpensive portable “mini” MS systems currently under development [43].

## Conclusions

Results of this study indicate that serum CFP-10 measurement can greatly improve TB diagnosis rates in HIV/TB-co-infected patients, who are diagnosed with reduced efficiency by current TB diagnostics. Improved TB diagnosis is a major unmet need for HIV-infected patients, as undiagnosed and untreated TB is a major cause of excess morbidity and mortality. In blinded assays performed with serum samples of HIV-infected patients, we found that serum CFP-10 diagnosed 89.6% of culture-positive and 66.7% of culture-negative TB cases, with 91.4% overall specificity. Results of this proof-of-concept study indicate that TB antigen detection has a strong potential to improve the speed and accuracy of TB diagnosis in HIV-infected patients.

## Additional files

Additional file 1: Figure S1. Schematic of the iPRM method analysis of CFP-10 from patient blood samples. **Figure S2**. Specimen distribution for HIV-infected tuberculosis diagnosis. (ZIP 410 kb)

Additional file 2: Table S1. Ion scanning and transition settings for LC-PRM MS analysis. (DOCX 45 kb)

Additional file 3: Table S2. Demographics and clinical characteristics of the study participants. (DOCX 48 kb)

## Abbreviations

AFB: Acid-fast bacilli
CDC: Centers for Disease Control and Prevention
CFP-10: Culture filtrate protein 10
DSHS: Texas Department of State Health Services
EPTB: Extrapulmonary tuberculosis
HTI: Houston Tuberculosis Initiative
MPB-64: Immunogenic protein MPB64
iPRM: Immunoaffinity-based parallel reaction monitoring
ESAT-6: 6 kDa early secretory antigenic target
MGIT: Mycobacteria growth indicator tube
Mtb: Mycobacterium tuberculosis
NTM: Non-tuberculous mycobacteria
PTB: Pulmonary tuberculosis
TB: Tuberculosis
TST: tuberculin skin test
WHO: World Health Organization
