# Supplementary material for: Rapid diagnosis of new and relapse tuberculosis by quantification of a circulating antigen in HIV-infected adults in the Greater Houston metropolitan area
Source: BMC Med. 2017 Nov 1;15:188. doi: 10.1186/s12916-017-0952-z (PMC5664577; doi:10.1186/s12916-017-0952-z)
Supplement: Supplementary file 1 — Schematic of the iPRM method analysis of CFP-10 from patient blood samples. Figure S2. Specimen distribution for HIV-infected tuberculosis diagnosis. (ZIP 410 kb) [file 12916_2017_952_MOESM1_ESM.zip › Supplementary Figure S2_renumber.docx]

**Figure S2.** Specimens resources distribution for HIV infected tuberculosis diagnosis
