# Supplementary material for: Rapid diagnosis of new and relapse tuberculosis by quantification of a circulating antigen in HIV-infected adults in the Greater Houston metropolitan area
Source: BMC Med. 2017 Nov 1;15:188. doi: 10.1186/s12916-017-0952-z (PMC5664577; doi:10.1186/s12916-017-0952-z)
Supplement: Supplementary file 1 — Schematic of the iPRM method analysis of CFP-10 from patient blood samples. Figure S2. Specimen distribution for HIV-infected tuberculosis diagnosis. (ZIP 410 kb) [file 12916_2017_952_MOESM1_ESM.zip › Supplementary Figure S1R2.docx]

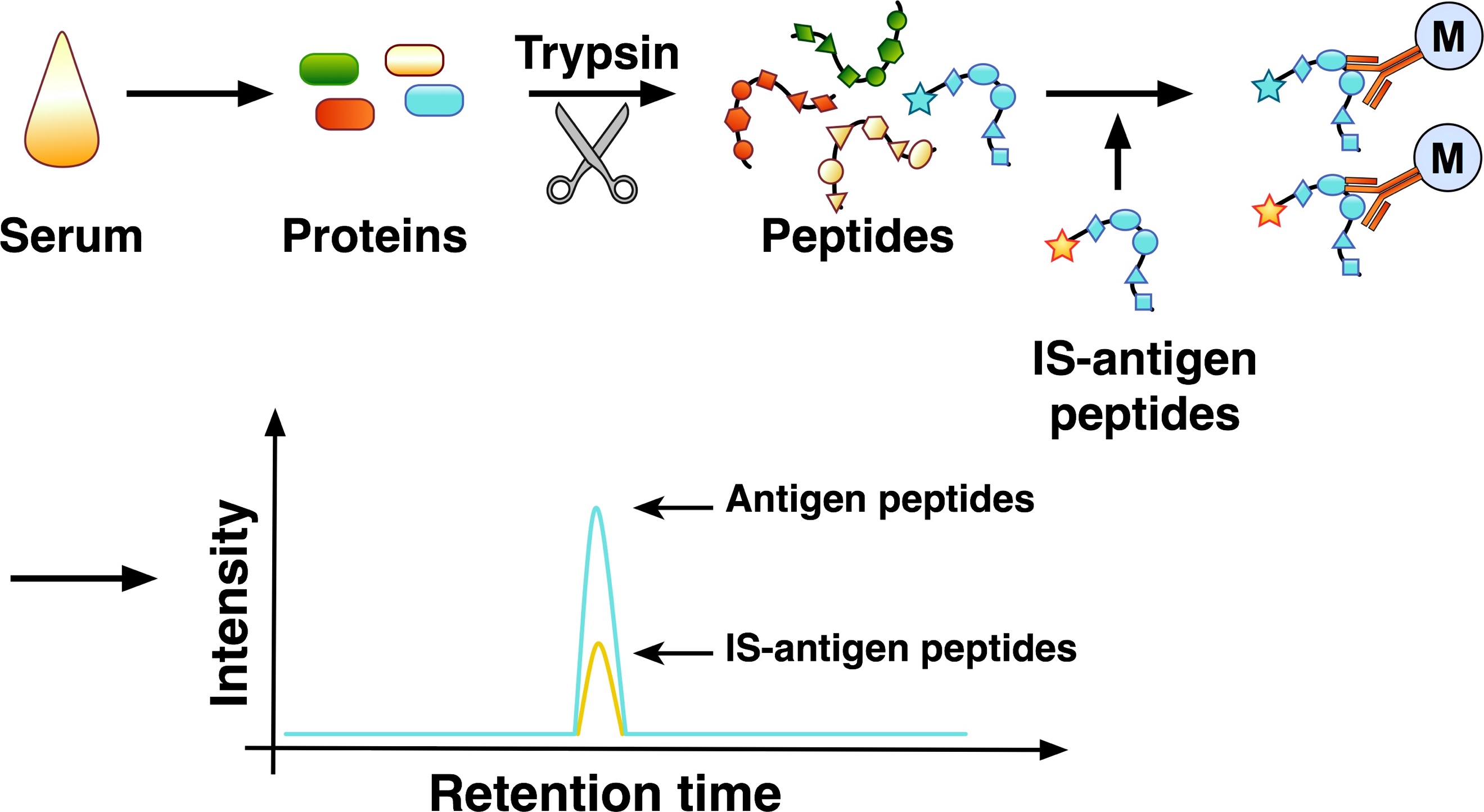


**Figure S1.** Schematic of the iPRM method analysis of CFP-10 from patient blood samples. Serum or plasma samples are trypsin digested to generate peptide fragments and then spiked with a stable-isotope-labeled internal standard peptide matching the target peptide sequence. Target and internal standard peptides are then precipitated with an antibody specific for the CFP-10 target peptide and analyzed by mass spectrometry, using the internal standard peptide signal intensity and known concentration to quantitate the target peptide. Both peptides have the same retention time due to their identical sequence, but the internal standard has a defined mass-to-charge ratio offset due to the inclusion of a fixed number of stable-isotope-labeled amino acids.
