# Supplementary material for: Rapid diagnosis of new and relapse tuberculosis by quantification of a circulating antigen in HIV-infected adults in the Greater Houston metropolitan area
Source: BMC Med. 2017 Nov 1;15:188. doi: 10.1186/s12916-017-0952-z (PMC5664577; doi:10.1186/s12916-017-0952-z)
Supplement: Supplementary file 2 — Ion scanning and transition settings for LC-PRM MS analysis. (DOCX 45 kb) [file 12916_2017_952_MOESM2_ESM.docx]

**Table S1.** Ion scanning and transition settings for LC-PRM MS analysis
