# Supplementary material for: Rapid diagnosis of new and relapse tuberculosis by quantification of a circulating antigen in HIV-infected adults in the Greater Houston metropolitan area
Source: BMC Med. 2017 Nov 1;15:188. doi: 10.1186/s12916-017-0952-z (PMC5664577; doi:10.1186/s12916-017-0952-z)
Supplement: Supplementary file 3 — Demographics and clinical characteristics of the study participants. (DOCX 48 kb) [file 12916_2017_952_MOESM3_ESM.docx]

**Table S2.** Demographics and clinical characteristics of the study participants

| **Cured TB** | **Recurrent TB** | **Cured TB** | **p value** |
| --- | --- | --- | --- |
| **Age:** mean (range) | 43 (35-50) | 40 (34-48) | 0.936 |
| **Male sex:**  # (%) | 8/9 (88.9%) | 12/15 (80.0%) | 0.572 |
| **Race** |  |  | 0.465 |
| Black: # (%) | 6 (66.7%) | 12 (80.0%) |  |
| White: # (%) | 3 (33.3%) | 3 (20.0%) |  |
| **CD4 T-cells/µL** (IQR) | 99 (31-446) | 324 (177 - 686) | 0.106 |
| **Log_10_ HIV copies/mL**  mean (range) | 4.1 (3.1-5.1) | 3.4 (2.6-5.0) | 0.317 |
| **Years of follow-up**  mean (range) | 7 (4.5-18) | 3 (2-4) | 0.004 |
